# Kynurenine Aminotransferase Isozyme Inhibitors: A Review

**DOI:** 10.3390/ijms17060946

**Published:** 2016-06-15

**Authors:** Alireza Nematollahi, Guanchen Sun, Gayan S. Jayawickrama, W. Bret Church

**Affiliations:** Group in Biomolecular Structure and Informatics, Faculty of Pharmacy, University of Sydney, Sydney, NSW 2006, Australia; gsun2866@uni.sydney.edu.au (G.S.); gjay8197@uni.sydney.edu.au (G.S.J.)

**Keywords:** kynurenine aminotransferase, pyridoxal 5′-phosphate, cognitive disorders, schizophrenia, KYNA

## Abstract

Kynurenine aminotransferase isozymes (KATs 1–4) are members of the pyridoxal-5’-phosphate (PLP)-dependent enzyme family, which catalyse the permanent conversion of l-kynurenine (l-KYN) to kynurenic acid (KYNA), a known neuroactive agent. As KATs are found in the mammalian brain and have key roles in the kynurenine pathway, involved in different categories of central nervous system (CNS) diseases, the KATs are prominent targets in the quest to treat neurodegenerative and cognitive impairment disorders. Recent studies suggest that inhibiting these enzymes would produce effects beneficial to patients with these conditions, as abnormally high levels of KYNA are observed. KAT-1 and KAT-3 share the highest sequence similarity of the isozymes in this family, and their active site pockets are also similar. Importantly, KAT-2 has the major role of kynurenic acid production (70%) in the human brain, and it is considered therefore that suitable inhibition of this isozyme would be most effective in managing major aspects of CNS diseases. Human KAT-2 inhibitors have been developed, but the most potent of them, chosen for further investigations, did not proceed in clinical studies due to the cross toxicity caused by their irreversible interaction with PLP, the required cofactor of the KAT isozymes, and any other PLP-dependent enzymes. As a consequence of the possibility of extensive undesirable adverse effects, it is also important to pursue KAT inhibitors that reversibly inhibit KATs and to include a strategy that seeks compounds likely to achieve substantial interaction with regions of the active site other than the PLP. The main purpose of this treatise is to review the recent developments with the inhibitors of KAT isozymes. This treatise also includes analyses of their crystallographic structures in complex with this enzyme family, which provides further insight for researchers in this and related studies.

## 1. Introduction

Kynurenine aminotransferase (KAT) isozymes, biologically active as homodimers, belong to the PLP (pyridoxal-5’-phosphate)-dependent enzyme family (PLP is covalently linked to the lysine residue of the enzyme through the Schiff base trans-aldimine linkage). These enzymes produce a crucial neuroactive compound, kynurenic acid (KYNA), through an irreversible transamination of a metabolite along the tryptophan catabolic pathway, named kynurenine (KYN) [1,2,3]. The enzymes accomplish their function at their active sites, which is at the cleft of the dimer interface. They also require an α-ketoacid as a co-substrate (providing the amino group acceptor in the PLP regenerating step) and PLP as a cofactor. The conversion of KYN to KYNA consists of two distinct steps. In the first step KATs catalyse KYN to an unstable intermediate, and in the next step, the intermediate product forms KYNA through a rapid cyclization reaction [4]. To date, four members of this family have been reported [5]. Among them, *Homo sapiens* crystal structures of KAT-1 and 2 have been deposited on the Protein Data Bank (PDB) server [6,7,8,9,10,11,12], along with one human model, published in 2016, for KAT-4 [13]. For KAT-3, only a *Mus musculus* crystallographic model has been reported [14]. The hKAT-1 enzyme is a 422 residue protein with one modified residue, LLP247, due to the internal aldimine linkage of PLP with LYS247 [15]. The aminotransferase activity is not only limited to kynurenine as a substrate, as, for example, glutamine is one of the well-studied substrates that was reported by Meister and colleagues in the 1950s and further confirmed by Cooper and Meister in the 1970s [16]. Moreover, Cooper *et al*. in 2016 showed glutamine to be favoured over kynurenine as a substrate *in vivo* [17]. The optimum pH for hKAT-1 activity is reported to be between eight and nine, and the more commonly-used α-ketoacid is pyruvate, although its efficiency is not the highest [18]. Likewise, hKAT-2, the second isoform of the KAT family, shows transamination activity with several amino acids. Each monomer has 425 residues, and it is the LYS263 of hKAT2 that is covalently bound to the PLP [19]. The most suitable α-ketoacid is α-ketoglutarate, as it has the highest activity observed in the assay of hKAT-2, with the optimum pH around seven [8,20]. KAT-3, the next isoform discovered in this enzyme family, shows the highest identity and similarity to KAT-1 [21]. Their resemblance is therefore also considered to be substantial, but there is no crystallographic study on hKAT-3. Optimum conditions for the activity assay of KAT-3 were recently obtained using the mouse enzyme. Among the α-ketoacid substrates studied for mKAT-3 activity, the more commonly-used ones are α-ketobutyrate and oxaloacetate. The reported optimum pH of mKAT-3 is nine [14,21]. KAT-4 is the last member of KATs, and though not extensively characterized yet, one crystal structure of hKAT-4 has been deposited in the PDB [13]. The biochemical conditions from rat brain KAT-4 proposed the optimum pH at around eight, which is almost consistent with the data for hKAT-4 with optimum activity at pH 8.5. The range of α-ketoacids used in the studies was on rat and mouse KAT-4, and most of them were suitable; but the group the worked on hKAT-4 used α-ketoglutarate as a co-substrate alone [5,13].

KYNA has been found in both peripheral tissues and brain, but its production and function in peripheral tissues has been significantly less studied. The role of KYNA, its biosynthesis and concentration in the human brain however has been intensively studied. It is known that KYNA has unique effects on several targets in the human central nervous system, and it is involved in many psychological conditions, such as neurodegenerative disorders, as well as cognitive impairment illnesses [22,23,24]. Our main interest is the activities of the KATs in the human brain, and it is well established that the most abundant of them is KAT-2 (60%). KATs perform their activities in the astrocyte cells [25,26]. Also important to introduce here, it is difficult for KYNA to passively cross the blood brain barrier (BBB), though a single study has shown a carrier, which is involved in the transportation of probenecid, can also transport KYNA through the BBB [27]. A significant increase in KYNA levels has been observed in cerebrospinal fluid (CSF) and brain samples of patients with schizophrenia [28]; hence this rise is inexorably linked to the KAT activity in human brain. A direct strategy to decrease the level of KYNA in human brain is the inhibition of KAT isozymes [29]. Human KAT-1 and 2 inhibitors are the most studied of them, but due to the predominant role of KAT-2 in the human brain, recent studies mainly focused on specific inhibitors of it. Although the first generation of KAT-2 inhibitors was reversible [30], the most potent ones were irreversible via a mechanism of permanent covalent bonding to LLP in the binding site of KAT-2 [31,32]. Studies reporting about KAT-2 inhibitors have been limited to *in vitro* evaluations, and *in vivo* considerations of the potential toxicity of the irreversible inhibitors have not yet been addressed. There have been systematic and comprehensive reviews on the kynurenine pathway [3,22,33,34], but there is no review that focuses on the inhibitors from different chemical classes and describing their probable mechanism of action. An important goal of this paper is to indicate the main pharmacophore and structural features of inhibitors of the KATs, accompanied by scrutiny of the structural aspects of the binding of the inhibitors and key interactions observed in the available crystallographic models.

## 2. Kynurenine Aminotransferases Inhibitors

As a start to this section, it is worth reinforcing that IC_50_ is a measure related to the concentration of the enzyme or receptor (generally known as a target), inhibitor and substrate together with many other conditions that define the assays. Sometimes IC_50_ values are used to compare potency and therefore mislead researchers in reaching their conclusions. These values are not able to clearly define the potency and selectivity properties of inhibitors, especially the irreversible ones. Another aspect that shows that the use of IC_50_ values in comparisons of inhibitors is not a good choice, is the role of the time of assay (exposure time), which plays a key role for both reversible and irreversible inhibitors. Due to such issues, other technical problems and the lack of standard protocols to evaluate and compare compounds, recently, emphasis has changed toward using the *K*_i_, which is independent from the amount of substrate [35,36]. Therefore, the IC_50_ values reported here are from different techniques and conditions, but the main goal is a focus on the structural features of these inhibitors.

**Figure 1:**
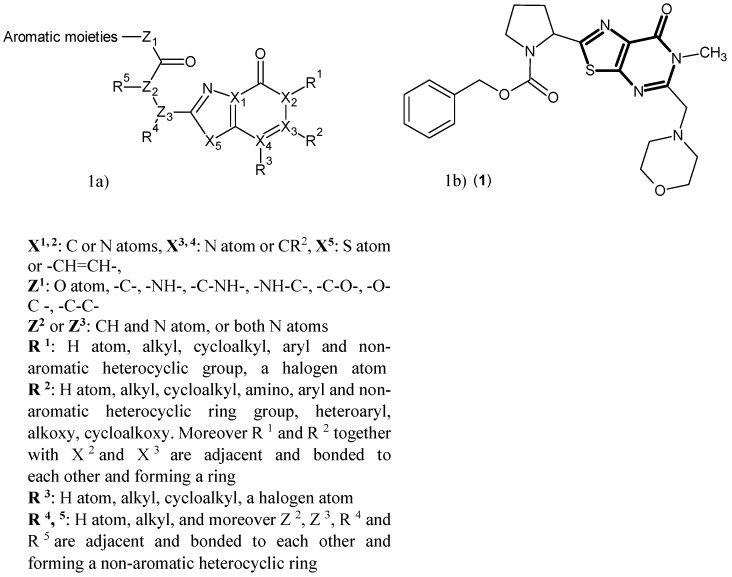
(**a**) Structures of the designed hKAT-2 inhibitors; (**b**) structure of Compound (**1**) as the most potent of these series of compounds, with the main pharmacophore in bold.

A series of heterocyclic compounds were synthesized in 2015, which represented considerable inhibitory activity against hKAT-2. The general structure of these compounds is shown in Figure 1a. From the several possibilities for the heterocyclic rings provided in Figure 1a, the optimum ring system is (1,3)thiazolo(5,4-d)pyrimidin-7(6H)-one (bold in Figure 1b). The most potent inhibitor (**1**) is shown in Figure 1b with a reported IC_50_ of 0.61 μM [37]. Compound (**1**) has six free rotatable bonds, which presumably allow the compound to be accommodated in the binding site more advantageously, with its nine hydrogen bond acceptors sites enhancing the binding affinity in the interaction pocket, compared to other designed inhibitors.

**Figure 2:**
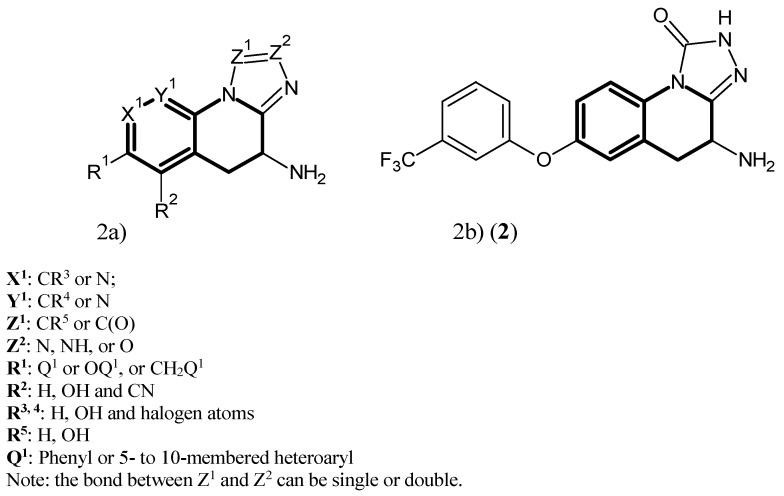
(**a**) Heterocyclic hKAT-2 inhibitors; (**b**) structure of Compound (**2**), being the most potent one. The main pharmacophore of the agents is in bold.

In work from Pfizer, the focus shifted to the inhibition of hKAT-2 aside from hKAT-1. Here, the core structure, shown in Figure 2a, was selected as a lead, and derivatives were synthesized. The best potency belonged to (**2**), depicted in Figure 2b, with the value of IC_50_ at 91.4 nM [38].

**Figure 3:**
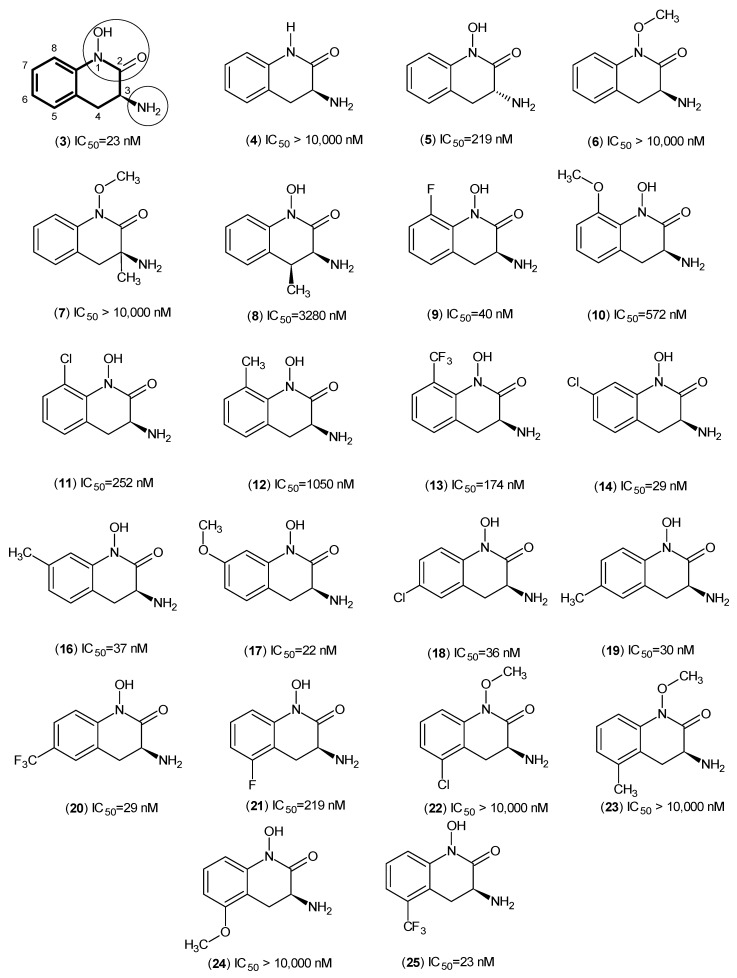
PF-04859989 (**3**) structure and its derivatives as hKAT-2 inhibitors. The main pharmacophore of PF-04859989 is in bold, and the essential structural elements are indicated by circles (keto, hydroxyl and amino groups).

In a related approach, the same Pfizer team published further work, which utilized a similar core structure, and they derived (**3**), PF-04859989 (Figure 3), another compound containing the core of dihydroquinolin-2-one that acts as an irreversible inhibitor of hKAT-2. From ^13^C NMR studies, it was shown that the amino group of (**3**) makes an irreversible aldimine bond with the PLP, which deactivated it permanently. The reported IC_50_ was approximately 23 nM [32].

The Pfizer team observed that the presence of a hydroxyl group at position 1 plays a key role in the activity of this series of compounds, and (**6**) confirmed that this free hydroxyl moiety is necessary because it can play a role as both a hydrogen bond acceptor and a donor. By looking at the biological data of (**3**) and (**5**), the stereoselectivity role of the amino group at position 3 was revealed. At position 3, the free amino group (which points upward, *S* configuration) is the most suitable moiety, and Compounds (**7**) and (**8**) indicated that no other small groups close to the amino moiety can be tolerated in the binding pocket because of the clash with the external aldimine linkage situated between the free amino group and PLP. The effects of different groups on the aromatic ring are discussed here due to the extensive investigations performed on the substitutions on the non-aromatic ring of the template. By looking at Compounds (**9**–**12**) and (**22**–**25**), it can be concluded that only small groups, like fluorine, are allowed at positions 5 and 8. Meanwhile, the biological assay results revealed that hKAT-2 has a large pocket, which not only provided enough room for substituents at positions 6 and 7, but one suitable for different sizes to be accommodated. To draw conclusions about the impact of altering the electronic properties of the moieties, more studies are required.

**Figure 4:**
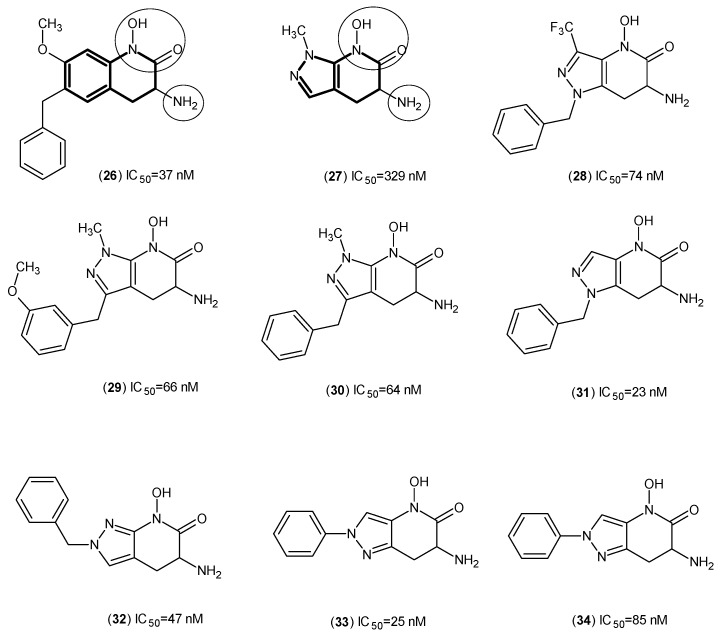
Pyrazole analogs as hKAT-2 inhibitors. The main pharmacophore of compounds are bolded and the essential structural elements are indicated by circles (keto, hydroxyl and amino groups).

In another study, again from the same team, (**3**) was employed as a template to design novel pyrazole-based inhibitors (Figure 4) [11].

Firstly, by use of biological assays, it was confirmed that Compound (**26**), based on the Compound (**3**) template position 6, can tolerate a benzyl group very well, and by selecting Compound (**27**) as a lead scaffold for the pyrazole series, other pyrazole inhibitors (**28**–**34**) were synthesized and evaluated. The results for Compound (**28**) backed up their hypothesis, and in comparison with Compound (**27**), the benzyl moiety enhanced the potency five times. By consideration of the other compounds, it can be concluded that the bioisosteric alteration to pyrazole would not affect the potency significantly, and even in Compound (**31**), the potency was improved. Moreover, it was observed that extra moieties on the pyrazole ring can be better tolerated than observed with the PF-04859989 template (e.g., Compound (**12**)). To sum up, the pyrazole series increase the chance of having improved interactions with the binding site of hKAT-2, but due to the universal presence of the free amino group at position 3 on the non-aromatic ring, the mechanism of action (covalent bond to PLP) is duly established.

**Figure 5:**
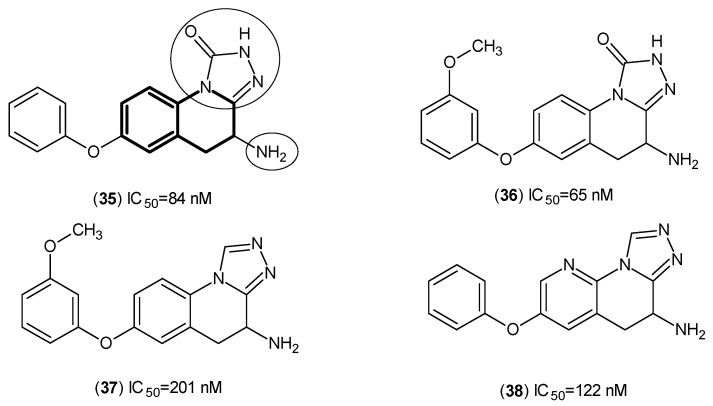
Designed and synthesized hydroxamate series as hKAT-2 inhibitors. The main pharmacophore of compounds is in bold, and the essential structure elements are indicated by circles (triazole-3-one ring and amino groups).

Further, to increase the prospects for PF-04859989 to have good brain penetration, the synthesis of a hydroxamate series was undertaken. However, such changes cannot mitigate any irreversibility issue with all of the synthesized compounds (Figure 5) [39].

**Figure 6:**
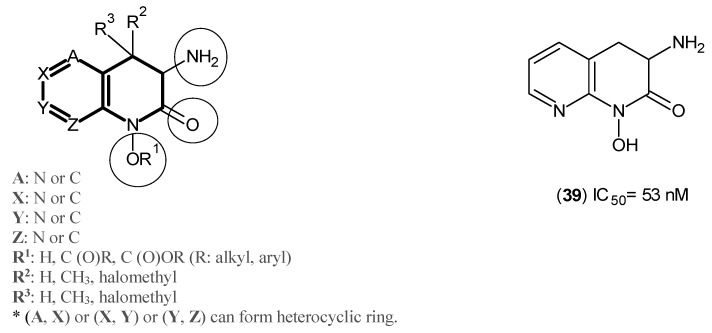
Designed hKAT-2 inhibitors. The main pharmacophore of compounds is in bold, and the essential structural elements are indicated by circles (keto, OR^1^ and amino groups).

Furthermore, from yet another comprehensive study on the same series of inhibitors from Pfizer, several compounds sharing the same nucleus depicted in Figure 6 were evaluated against hKAT-2, and from this, Compound (**39**) was determined to be the most potent with an IC_50_ of 53 nM [40].

**Figure 7:**
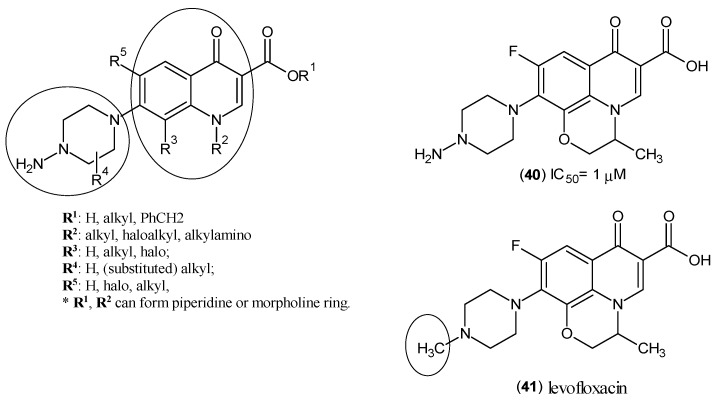
Structural nuclei of hKAT-2 inhibitors and structures of BFF-122 (**40**) and levofloxacin (**41**). The essential structural elements are indicated by circles (piperazine and heterocyclic rings). A circle on (**41**) highlights the only difference between BFF-122 and levofloxacin, where there is a methyl group.

Another approach focusing on hKAT-2 inhibitors yet again resulted in a new promising lead nucleus shown in Figure 7. These groups of inhibitors, of which fluoroquinolones are a subset, are known antimicrobial agents. The amino group on the piperazine ring indicated the mechanism of action of irreversible inhibition via the interaction with PLP. The moieties of the synthesized compounds are shown in Figure 7, and these structures presented potencies (IC_50_) at between 1 and 2 μM [41]. One of the well-studied compounds of this family is BFF-122 (Compound (**40**)) with an IC_50_ value of 1 μM [9]. It is of interest that this compound shares high similarity with levofloxacin (**41**) (Figure 7), which suggests that BFF-122 should be closely scrutinised due to the well-established severe adverse effects of levofloxacin, like tendon rupture and inflammation [42], convulsion [43], psychosis [44] and possibly permanent peripheral neuropathy [45].

**Figure 8:**
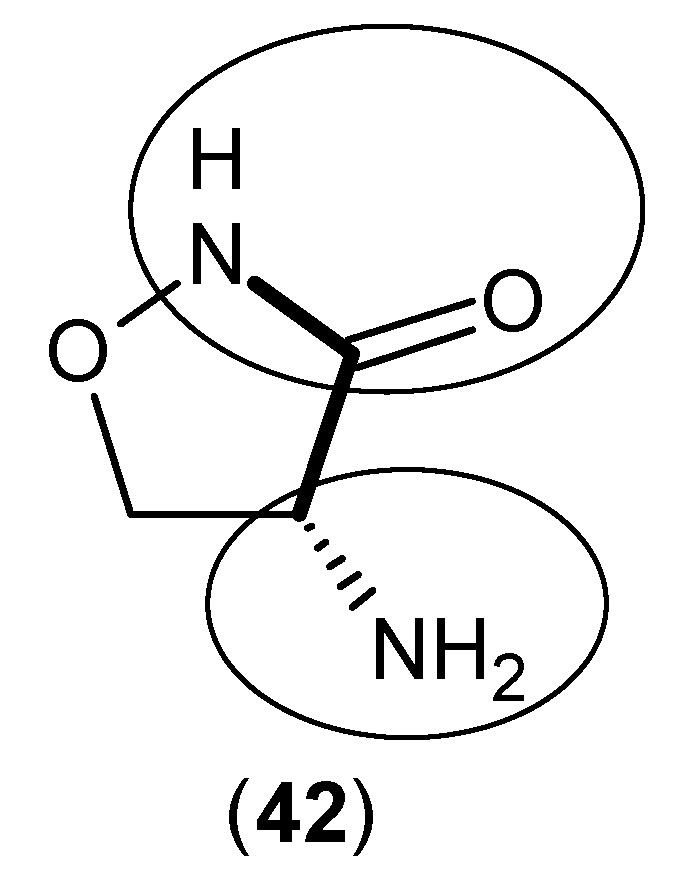
Structure of d-cycloserine (**42**), with its main pharmacophore in bold, and the essential structural elements are indicated by the circles (keto and amino groups).

In studies in which clinical trials were undertaken, it was shown that d-cycloserine, Compound (**42**) (Figure 8), has positive effects of enhancing cognitive functions in patients with schizophrenia or those additionally experiencing Alzheimer’s delusions, by decreasing KYNA levels in the human brain by inhibiting all of KAT-1, 2 and 3. The reported inhibitory activities of this compound against KAT-1, 2 and 3 in the human frontal cortex were 54, 66 and 72 at 64 μM, respectively [46]. From Compound (**3**), which was the template utilized, it was observed that instead of using the *R* configuration of the amino group, the *S* form may universally provide higher potency. Moreover, very early in the studies of these enzymes, back in 1979, the inhibitory effects of some antibacterial agents on rat KAT-1 reported the same results, where d-cycloserine (**42**) and novobiocin inhibited the rat KAT-1 activity [30].

**Figure 9:**
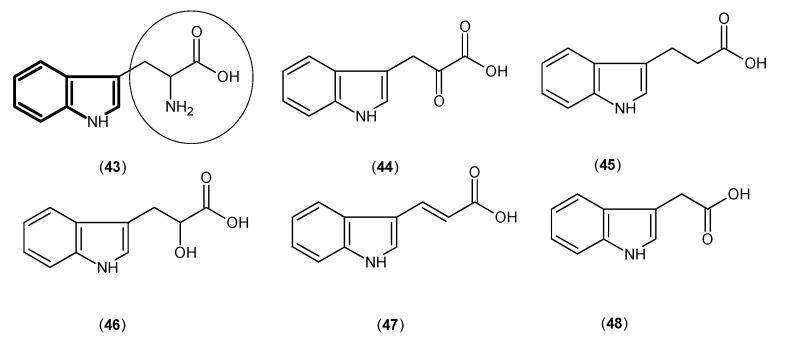
Structures of tryptophan (**43**) and indole derivatives as KAT-1 inhibitors. The main pharmacophore is in bold, and the essential structural elements are indicated by circles (carboxylic and amino groups).

As tryptophan (**43**) is the main precursor of kynurenine in the catabolic pathway, tryptophan and five other chemicals (Figure 9), which share the 1H-indole core and similar side chains to tryptophan, were used in a relatively basic approach and their inhibitory activity was evaluated against KAT-1. Some of these studies predate some of the KAT-2 studies already shown. Among them 3-indolepropionic acid (**45**) and ±-indole-3-lactic acid (**46**) represented the best inhibitory effects with values of IC_50_ 140 and 220 μM, respectively [7].

**Figure 10:**
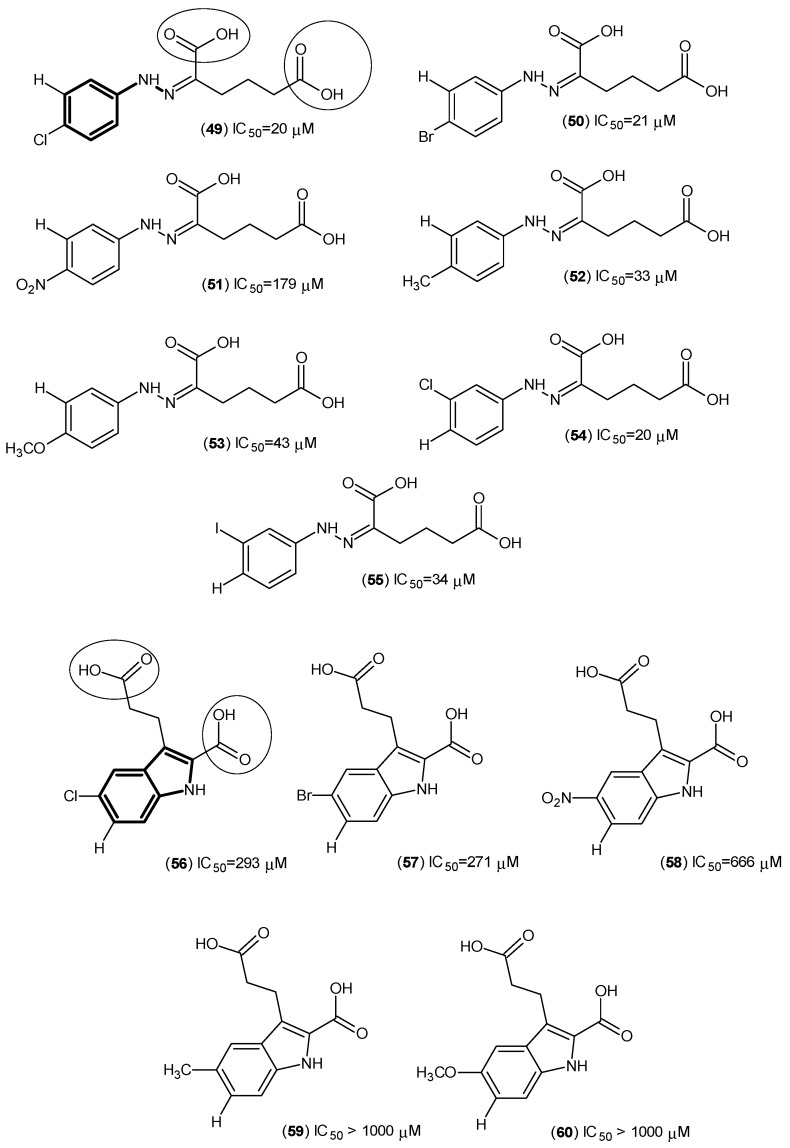
Structures of 1H-Indole-3-propanoic acid and hexanoic acid derivatives as hKAT-1 inhibitors. The main pharmacophore of these is in bold, and the essential structural elements are indicated by circles (carboxylic groups).

In a continuation study, another research team reported twelve 1H-Indole-3-propanoic acid and hexanoic acid derivatives (Figure 10) that were synthesized for the purpose of considering their inhibition of hKAT-1 [47].

The most potent synthesized compound belonged to the one with the hexanoic group, Compound (**49**), and was named 5-(2-(4-chlorophenyl) hydrazono)-6-ethoxy-6-oxohexanoic acid, with an IC_50_ of 20 μM [47]. It was seen that moieties on the phenyl ring are key to the modulation of the potency, such as the instances of the presence of a hydrophobic group, like chlorine, which provides the most potent case, and from the bromine derivatives, it was seen that the pocket can accommodate the most bulky of the groups; yet, on the other hand, electron withdrawing moieties, such as the nitro group, decrease the potency of inhibitors. Moreover, from the hexanoic acid derivatives, it appears that the flexibility of the rotation at the side chain was also part of a significant increase in the potency.

**Figure 11:**
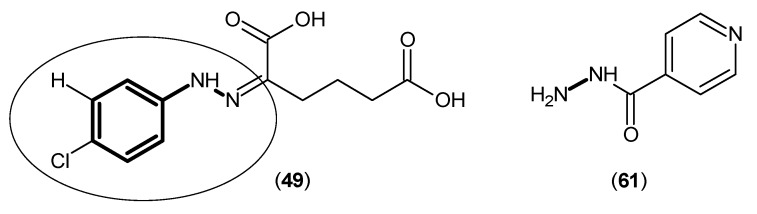
Structure of isonicotinic acid hydrazide (**61**) as an inhibitor of the KATs. The similarity between compounds (**49**), from hexanoic acid derivatives, and isonicotinic acid hydrazide are in bold and indicated by a circle.

One more aspect about Compound (**49**) is the existence of the phenylhydrazine nucleus. From much earlier work on possible antituberculosis agents and from an *in vivo* evaluation, it was reported that isonicotinic acid hydrazide (**61**) (Figure 11), also showed very promising inhibitory activity against KATs [48].

**Figure 12:**
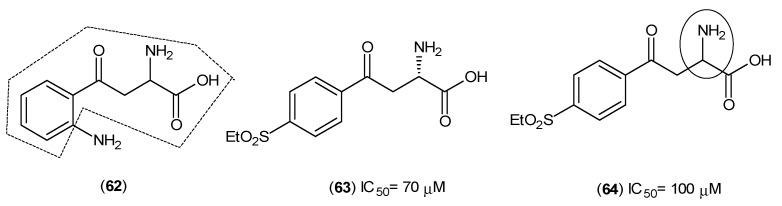
Structures of kynurenine (**62**), (*S*)-(4-ethylsulfonyl) benzoylalanine hydrochloride ((*S*)-ESBA) (**63**) and its racemic mixture (**64**). The form of kynurenine that was used in designing inhibitors is highlighted by a polygon. The amino group, which plays a key role in stereoselectivity of ESBA in racemic mixtures is indicated by a circle.

Using KYN, the natural substrate in the kynurenine pathway, Compound (**62**), as a template, a group of inhibitors retaining the benzoyl-alanine core were designed and tested as KAT-2 inhibitors. The first generation of a specific KAT-2 inhibitor was (*S*)-(4-ethylsulfonyl) benzoylalanine hydrochloride ((*S*)-ESBA) (**63**) (Figure 12). This compound was firstly reported in 2006 [49], and it features both selectivity and reversibility in inhibiting KAT-2 activities. The reported IC_50_ of this compound was around 1000 μM for hKAT-2, but not determined too precisely. As shown in Figure 12, Compound (**63**) exhibited higher potency and a reduced potency in its racemic mixture (**64**), which indicated the significance of stereoselectivity. Due to its activity, a fast way to synthesize (*S*)-ESBA and its derivatives was introduced later [50].

A study of hKAT-2 inhibition showed that Compound (**63**) is 20-times weaker compared to rat KAT-2. Even though a comparison of the sequences of the two enzymes shows that rat and human sequences share a high degree of similarity, the homology modelling studies suggest more flexibility in the arm in the N-terminal region (loop) of rat KAT-2, which allows easier access of this inhibitor into the binding site compared to hKAT-2 [51].

**Figure 13:**
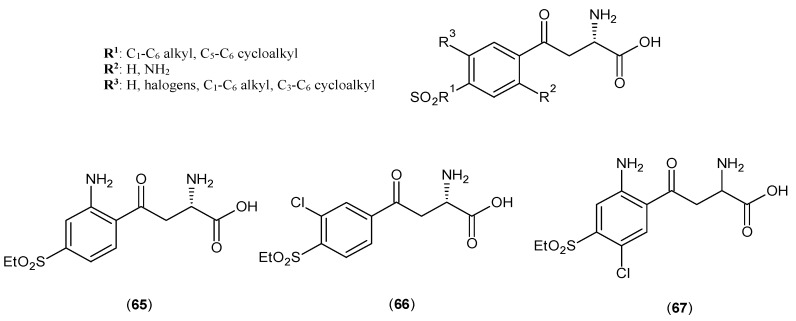
The core structure of 4-sulfonyl-substituted benzoylalanine derivatives acts as a KAT-2 inhibitor.

A study of 4-sulfonyl-substituted benzoylalanine derivatives considered as potential KATs inhibitors was reported in 2006 [52]. The core structure of these series of inhibitors is depicted in Figure 13. The inhibition bioassay was done on rat liver extractions only, and three compounds (**65**–**67**) showed promising IC_50_ values, and their ranges were between 2 and 25 μM.

**Figure 14:**
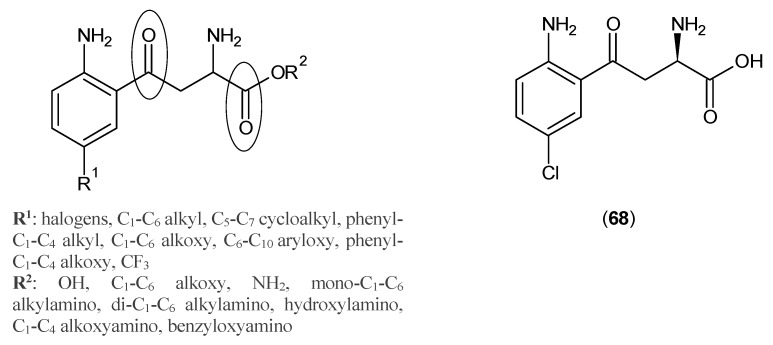
Kynurenine analogues as KATs inhibitors. The role of keto groups for interacting with active site residues of KATs are pointed out by circles.

In 1995, Schwarcz *et al.* [53] pursued a study of kynurenine analogues as KAT inhibitors. The nucleus they adopted for their study is depicted in Figure 14. Compound (**68**) showed around 80% inhibition of rat KAT-2 at 1000 μM [53]. The same team obtained IC_50_ of 5.4 μM for Compound (**68**) by evaluating its inhibition against rat KAT-2 in brain, which was almost five-times higher than the affinity of KYN to the enzyme. Moreover, they found that by omitting the keto group from the side chain, the inhibitory effect is reduced to almost negligible [54].

**Figure 15:**
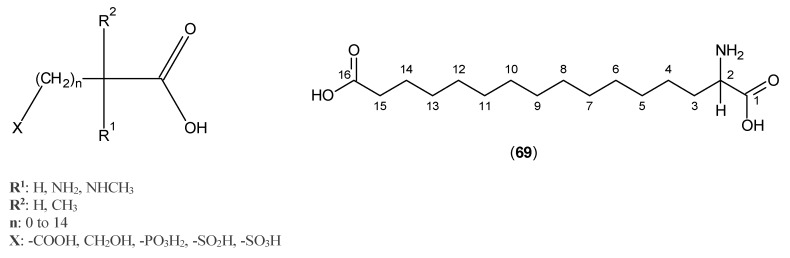
Dicarboxylic acid derivatives as KATs inhibitors.

Dicarboxylic acid derivatives were also examined for their inhibitory activity against KATs. The general structural formula of these compounds is shown in Figure 15, and among them, (±)-2-amino-1,6-hexadecanedioic acid, Compound (**69**), presented the highest activity with the value of IC_50_ 6.5 μM [55].

**Figure 16:**
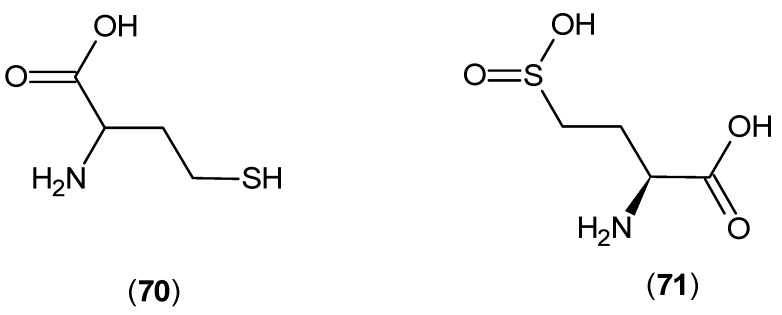
Cysteine analogs as KATs inhibitors.

In biochemical studies, KAT-1 and 2 were found to be expressed in the cytoplasm of bovine aortic endothelial cells, and the production of KYNA was monitored in the presence of ±-homocysteine (**70**) and l-homocysteine sulfinic acid (**71**) (shown in Figure 16); the IC_50_ results of 54 and 100 μM, respectively [56], were encouraging.

**Figure 17:**
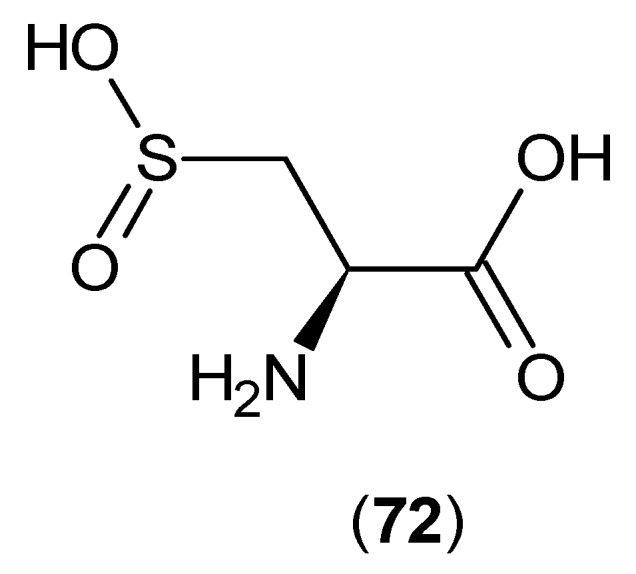
Structure of l-cysteine sulfinic acid (**72**) as a KAT-1 and 2 inhibitor.

These results are consistent with the outcomes from other studies on the effects of l-cysteine sulfinic acid (**72**) (Figure 17) on the inhibition of rat brain KAT-1 and 2, and the reported IC_50_’s were 80 and 20 μM, respectively [57]. Therefore, from these two studies, it can be considered that these endogenous sulphur-containing amino acids could possibly have modulatory activities in the production of KYNA.

**Figure 18:**
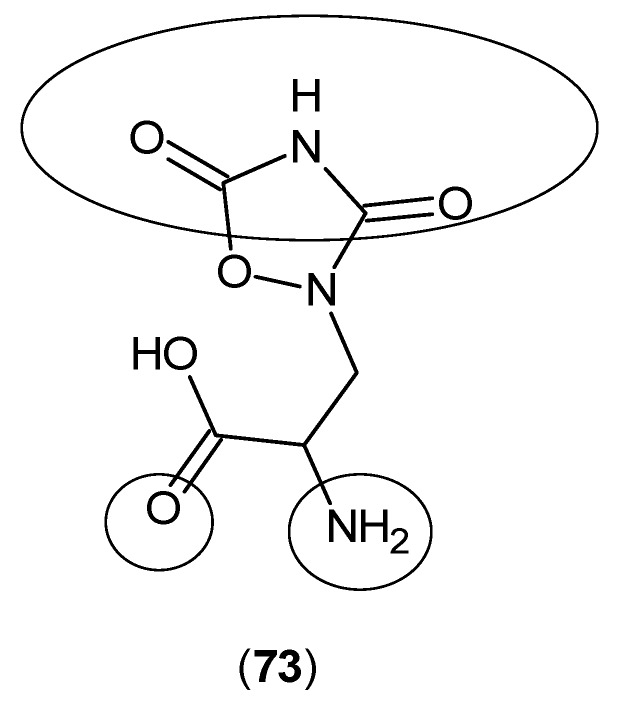
Structure of quisqualate (**73**), a known glutamate receptor agonist, as a KAT-1 and 2 inhibitor. The keto and amino groups, which have a predominant role in the interaction with the KAT active site residues, are pointed out by circles.

Furthermore, a group of simple glutamate receptor ligands was examined for inhibitory activities against KAT-1 and 2, and it was shown that quisqualate (**73**) (Figure 18) is able to inhibit the production of KYNA [58].

**Figure 19:**
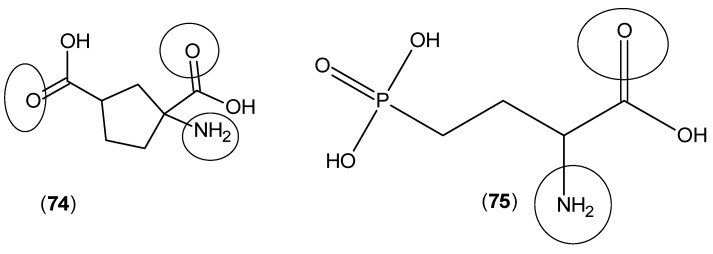
Structures of 1-aminocyclopentane-1,3-dicarboxylic acid (**74**) and 2-amino-4-phosphonobutanoic acid (**75**). The keto and amino groups, which have a predominant role in the interaction with KAT active site residues, are highlighted by circles.

In a continuation, the same research team considered the other metabotropic glutamate receptor agonists, such as 1-aminocyclopentane-1,3-dicarboxylic acid (**74**) and 2-amino-4-phosphonobutanoic acid (**75**) (shown in Figure 19), and all of them reduced KYNA production in rat brain tissues [59].

**Figure 20:**
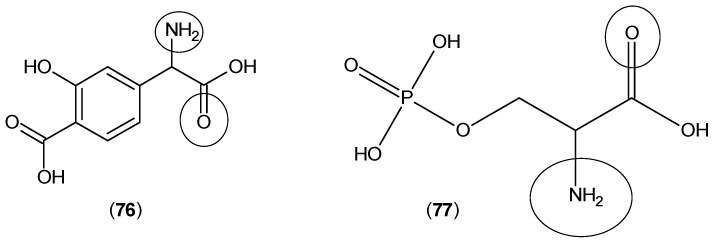
Structures of 4-(amino(carboxy)methyl)-2-hydroxybenzoic acid (**76**) and 2-amino-3-(phosphonooxy)propanoic acid (**77**). The keto and amino groups, which have a predominant role in the interaction with KAT active site residues, are highlighted by circles.

In a more extensive approach on the same compounds and other agonists of these types of receptors, a different research team confirmed the previous results. Compounds (**74**) and (**75**) along with 4-(amino(carboxy)methyl)-2-hydroxybenzoic acid (**76**) and 2-amino-3-(phosphonooxy)propanoic acid (**77**) (Figure 20) showed inhibitory activities against rat brain KAT-2 and decreased the levels of KYNA, while these compounds did not show any activity against KAT-1. This research team finally suggested that numerous metabotropic glutamate receptor agonists are able to reduce the production of KYNA by an intracellular mechanism in which the inhibition of the KYN transamination occurs by blocking KAT-2 activity [60].

**Figure 21:**
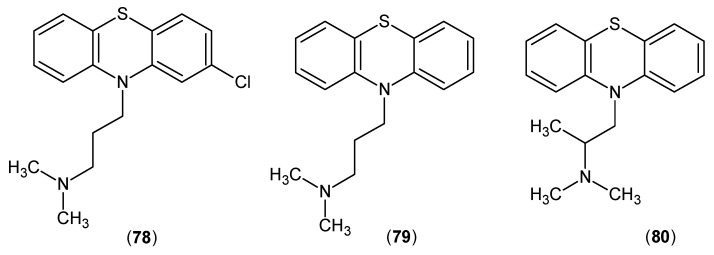
Structures of chlorpromazine (**78**), promazine (**79**) and promethazine (**80**), which inhibited the KATs *in vivo*.

In 1982, the effects of several known antipsychotic drugs were examined with the KATs. *In vivo* results revealed that KYNA production had a decrease after the inhibition of KAT activity by chlorpromazine (**78**), promazine (**79**) and promethazine (**80**), depicted in Figure 21. *In vitro* tests indicated that these compounds did not have any effects on the KATs [61].

**Figure 22:**
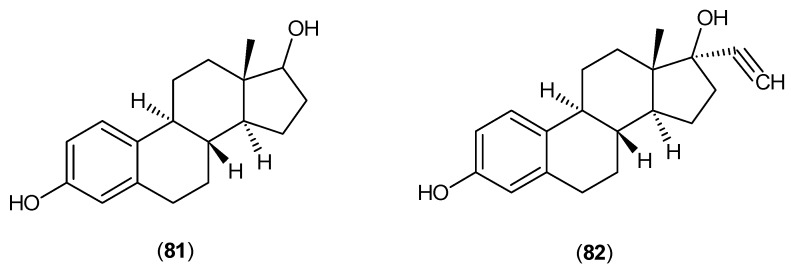
Structures of β-estradiol (**81**) and ethynylestradiol (**82**), which inhibited KATs in male mice.

Between 1959 and 1976, a small number of studies were done examining the effects of estradiol analogues on the activity of the kynurenine aminotransferase isozymes. One study showed that β-estradiol (**81**) and ethynylestradiol (**82**), shown in Figure 22, could inhibit the activity of male mice KATs [62].

**Figure 23:**
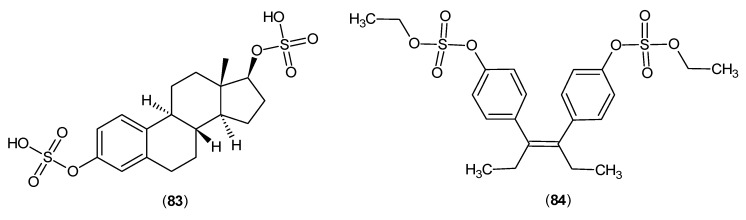
Structures of estradiol disulphate (**83**) and diethylstilbestrol disulphate (**84**), which inhibited the KATs.

In another study, the results demonstrated that conjugated estrogens affected the production of kynurenic and xanthurenic acid levels, two known products of KAT isozymes [63]. Moreover, estradiol disulphate (**83**) and diethylstilbestrol disulphate (**84**) (Figure 23) are able to inhibit KATs. It was proposed that these compounds are competitive and reversible inhibitors as their inhibitory effects are dependent on the concentration of the cofactor, PLP [64].

**Figure 24:**
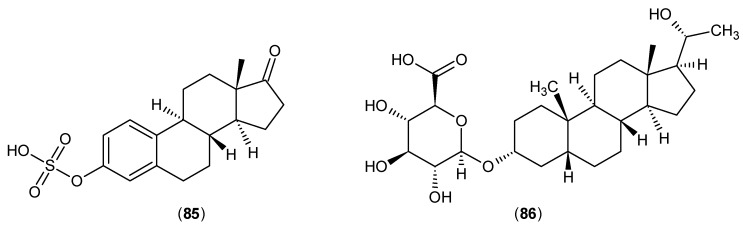
Structures of estrone sulphate (**85**) and pregnanediol glucuronide (**86**), which inhibited the rat KATs.

These results were consistent with the outcomes of research that followed on rat KAT activities in the presence of estradiol disulphate and diethylstilbestrol disulphate. Furthermore, the effects of estrone sulphate (**85**) and pregnanediol glucuronide (**86**) (Figure 24) were evaluated against rat KATs, and these two could inhibit this enzyme family in a similar manner at a slightly higher concentration in comparison with (**83**) and (**84**). The PLP was not implicated with a direct role in the mechanism of their inhibitory effects, as it was proposed that they act as reversible inhibitors and their actions are probably due to the interaction with other parts of the catalytic active site [65].

## 3. Interactions of Inhibitors and KATs

To provide a deeper understanding of key residues in the active sites of the enzymes involved in the interaction with inhibitors, this section examines the crystallographic structures in complex with inhibitors available at the PDB, as well as reviews and discusses them.

**Figure 25:**
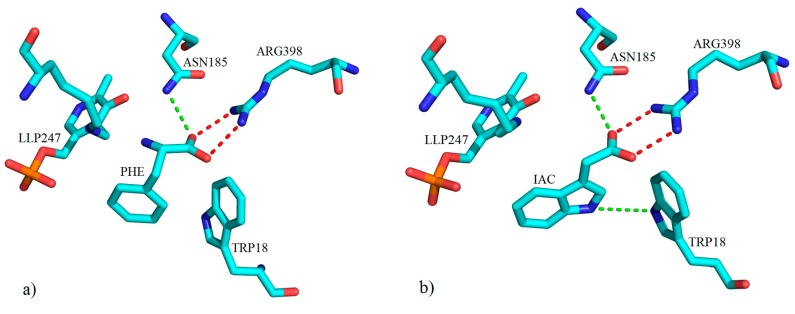
Depiction of the key molecular interactions between amino acid residues of the active site of hKAT-1 with (**a**) PHE (PDB code: 1W7M) and (**b**) IAC (PDB code: 3FVU) in the presence of LLP247 (PLP + LYS247). Both ligands form a hydrogen bond with ASN185 via their keto groups (green broken lines). A salt bridge interaction between ARG398 and the carboxylate moiety of both ligands is shown with red broken lines. All three-dimensional figures prepared using PyMOL [68].

The first crystal structure of hKAT-1 was deposited in the PDB in 2004 [66], along with a model of h-KAT-1 in complex with L-phenylalanine (PDB Entry 1W7M), which is a weak substrate compared to the natural substrate (KYN) and acts as a competitive inhibitor [67]. From these structural analyses, two residues, ASN185 and ARG398, were recognized to have important roles (Figure 25a), the roles of which were also borne out in a structure determined in 2009 (PDB Entry 3FVU) [7]. This was a crystal structure of hKAT-1 in complex with indole-3-acetic acid (IAC) (**48**). In both models, ASN185 and ARG398 had hydrogen bonds with the carboxylate groups of these two inhibitors, and therefore, the presence of keto groups on the side chain of inhibitors appeared crucial to having the inhibitory activity. Furthermore, in the 3FVU model, a further hydrogen binding site existed with TRP18 (Figure 25b), which can be a focus in the design of more potent inhibitors.

**Figure 26:**
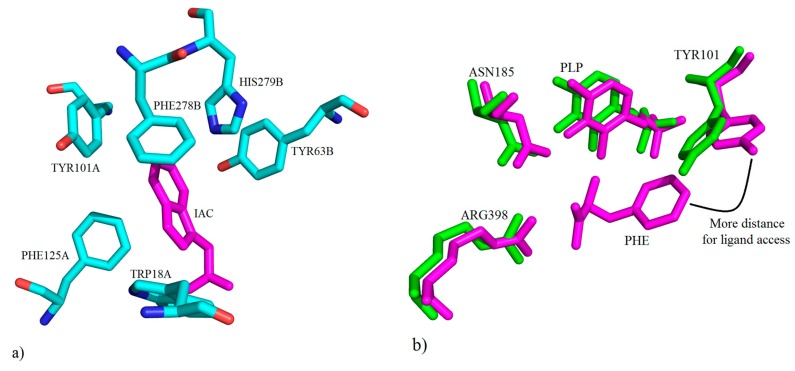
(**a**) Presentation of the key molecular interactions between amino acid residues of the active site of hKAT-1 involved in hydrophobic interactions with IAC (shown in magenta) (PDB code: 3FVU); (**b**) depiction of the native hKAT-1 structure (PDB code: 1W7L) superimposed on the structure of hKAT-1 in complex with PHE (PDB code: 1W7M) to show the conformational change of TYR101’s role.

There are hydrophobic interactions TYR101, PHE125 and TRP18 from one monomer and TYR63, PHE278 and HIS279 from the other subunit, which form the basis of the environment of the binding cavity (Figure 26a). Interestingly, it is the case that TYR101 is involved in a significant change at the binding site in hKAT-1 compared to other mammalian KAT-1s. TYR101 has two configurations (Figure 26b), one in the native state of hKAT-1 without inhibitor, also called the closed conformation, and the second in the presence of inhibitors or other ligands, known as the open conformation.

**Figure 27:**
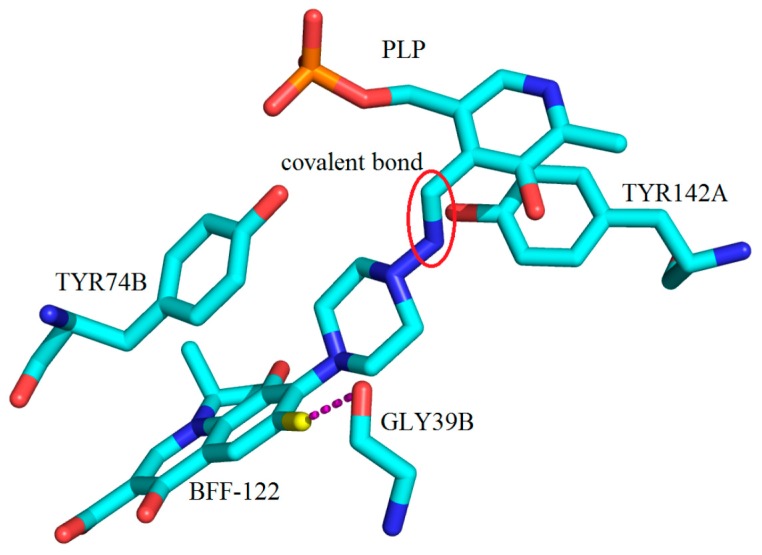
Key molecular interactions between amino acid residues of the active site of h-KAT2 involved in hydrophobic interactions with BFF-122, which is covalently bonded to PLP (cofactor) (indicated by a red circle); PDB code: 2XH1. The electrostatic interaction of the fluorine moiety of BFF-122 with GLY39B is highlighted by magenta broken lines.

In 2010, a crystal structure with BFF-122 (**40**) was reported in complex with hKAT-2 (PDB Entry 2XH1) along with the report of it as one the most potent hKAT-2 inhibitors [9]. Close analysis revealed that BFF-122 made a permanent covalent bond to PLP, the cofactor through the Schiff base reaction. This external aldimine linkage irreversibly deactivates the enzyme and so is a permanent end to the enzymatic activity. In the active site, the aromatic ring of TYR74 is observed to have a π–π interaction with the quinoline core of BFF-122, and also, GLY39 showed an electrostatic interaction with the fluorine moiety of BFF-122 (Figure 27).

**Figure 28:**
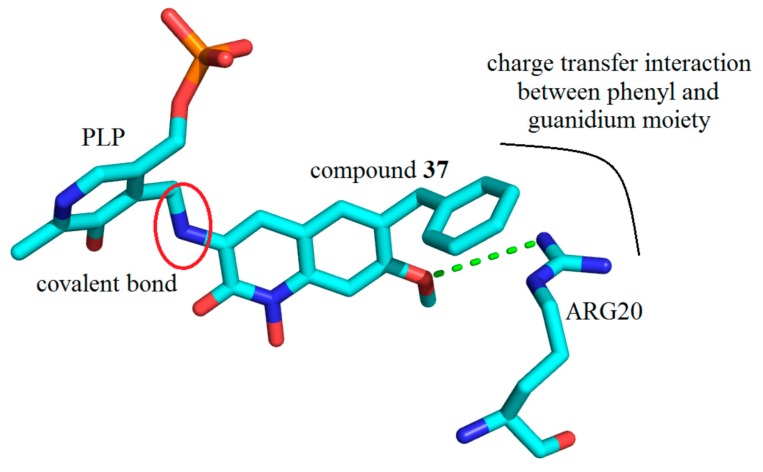
Key molecular interactions between ARG20 in the active site of hKAT-2 with Compound (**37**) covalently bonded to the PLP cofactor (indicated by the red circle); PDB code: 4GE9. Compound (**37**) forms a hydrogen bond with ARG20 via its methoxy moiety (green broken line).

It is obvious that the pocket of hKAT-2 can accommodate a bulky inhibitor very well, and this property of the hKAT-2 pocket is, in large part, due to the flexible loop, ARG20-SER32, which acts as an entrance gate; and the significant displacement it can undertake accommodates additional bulk. Similarly, the structure of hKAT-2 in complex with Compound (**37**) (PDB Entry 4GE9) showed a role for ARG20 specifically. The methoxy group at position 6 can form a hydrogen bond with ARG20, and due to this interaction, the guanidinium group in ARG20 also has a charge transfer interaction with the phenyl ring at position 7 (Figure 28). It is considered that with both the conformational change and guanidinium interaction, the free binding energy stability and potency are enhanced considerably [10].

**Figure 29:**
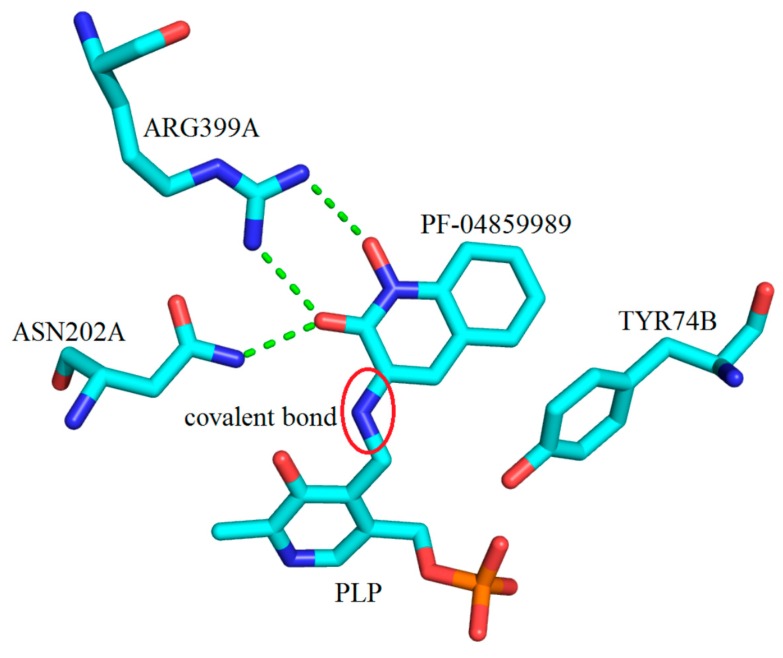
The three-dimensional depiction of key molecular interactions between amino acids of the active site of hKAT-2 with PF-04859989 covalently bonded to PLP (cofactor) (indicated by a red circle); PDB code: 3UE8. PF-04859989 forms hydrogen bonds with ARG399A and ASN202A through its methoxy and hydroxyl moieties, shown with green broken lines.

To continue, after the arrival of the potent hKAT-2 inhibitor, PF-04859989 (**3**), the crystal structure of this compound in complex with hKAT-2 revealed the same mechanism of action as with BFF-122. PF-04859989 made an irreversible covalent bond to PLP and permanently inactivated the enzyme. TYR233 and ASN202 both form hydrogen bonds with the PLP component of the complex (PF-04859989 + PLP), and ASP230 and TYR195 are involved in interactions with the pyridine ring of the PLP. The hydroxyl and keto moieties on the quinoline core of PF-04859989 make two key hydrogen bonds (as acceptor sites) with ASN202 and ARG399. Similar to the BFF-122 active site pocket, TYR74 again showed hydrophobic contact with PF-04859989 (Figure 29). It is worth commenting that by consideration of the hydrogen bond interactions with the core of the inhibitors for both the KAT-1 and 2 active sites, it can be concluded that the role of ASN185, ARG398 in hKAT-1 and the role of ASN202, ARG399 in hKAT-2 are the same. Therefore, the design of the KAT inhibitors should impose the presence of the keto groups in the nucleus.

**Figure 30:**
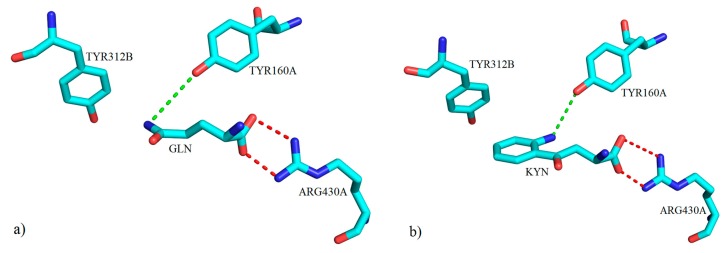
The three-dimensional representation of key molecular interactions between amino acid residues of the active site of mKAT-3 (**a**) with GLN (PDB code: 3E2Y) and (**b**) with KYN (PDB code: 3E2Z). Both ligands form a hydrogen bond with TYR160A via their amino groups (green broken lines). Salt bridge interactions between the ARG430A residue and the carboxylate moiety for both ligands are shown with red broken lines.

The only available crystal structure for KAT-3 is from mouse. As mentioned before, there is high similarity between hKAT-1 and mKAT-3; therefore, as expected, almost the same residues are present in the binding pocket of both hKAT-1 and mKAT-3 enzymes. From the sequence alignment, it is seen that the 1W7L entry for hKAT-1 will bear a high degree of similarity of structural features to that which will exist in the mKAT-3. The model of mKAT-3 in complex with glutamine, as a ligand, showed that several residues of the enzyme are involved in the binding, but among them, the guanidinium group of ARG430A forms hydrogen bonding and electrostatic interactions with the carboxylate moiety of the bound glutamine. Crystal structures of the mKAT-3 (the homodimer contains chains A and B) in complex with substrates (kynurenine and glutamine) revealed that TYR160A and TYR312B played a key role in the binding site (Figure 30), and TYR160A moves close to them to make a hydrogen bond, while TYR312B moves away from the cleft of the active site to supply enough space for substrates to have suitable contacts compared to the native form (Figure 30). The alignment of crystal structures also showed that the roles for PHE125A and PHE278B in hKAT-1 are equivalent to TYR160A and TYR312B in mKAT-3, respectively. Therefore, it is evident that the mKAT-3 active site is similarly more suitable for hydrophilic ligands compared to hKAT-1 [14]. Hence, it can be concluded that to design leads to inhibit KAT-3, the governing rules used for hKAT-1 as a starting point will suffice.

**Figure 31:**
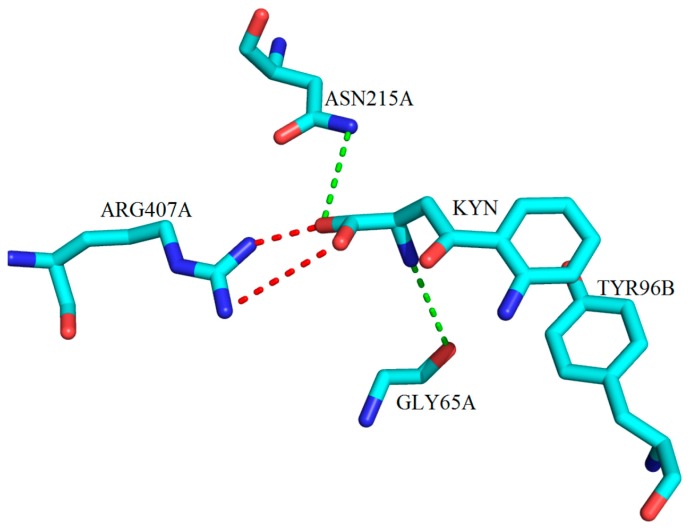
The three-dimensional depiction of key molecular interactions between amino acid residues of the active site of mKAT-4 with KYN (PDB code: 3PD6). KYN forms hydrogen bonds with GLY65A, TYR160A and ASN215A via its amino and keto groups, (green broken lines). Salt bridge interactions between the ARG407A and the carboxylate moiety of KYN are shown with red broken lines.

The structural work on the hKAT-4, provided in PDB Entry 5AX8, did not involve any complex with a substrate or an inhibitor [13]. Therefore, herein, we have been content to review only the mouse model of the KAT-4 enzyme. Again, the binding cup around the phosphate group on PLP (as described in [12]), as expected, followed the same structural architecture observed in the crystal structure in complex with the natural substrate (KYN). Examination of the binding pocked showed a salt bridge between ARG407 and the carboxylate of the substrate, plus a hydrophobic contact with TYR96 from the other subunit (Figure 31). The similarity between residues and other residues of KATs involved lead to good general design principles for the core of the inhibitors, but for selectivity, further investigation of the pocket size, shape and key residues will be necessary [69].

## 4. Conclusions

KAT isozymes are PLP-dependent enzymes and are very worthy of scrutiny as targets in the field of CNS drug development. Primarily, the interest is because this enzyme family is involved in the tryptophan catabolic pathway, one of the vital catabolic pathways in the human brain. This pathway also includes many other natural endogenous metabolites, which can act as inhibitors or as activators in different biochemical pathways of the brain. The presence of KATs in two separate parts of this pathway is evidence that they play a key role in keeping the balance for many of these endogenous chemicals, and so, their significant role is indisputable. This review has covered a significant proportion of the KATs inhibitors reported and has discussed them from the medicinal chemistry perspective, providing their probable mechanism of action. Unfortunately, there are not many *in vivo* studies reported for these compounds, even though there are serious claims of significant potency for them. Hence, further systematic *in vivo* examinations of these and related compounds are desirable. In summary, this review is presented in order to provide an in depth understanding of the existing inhibitors, along with a focus on the structural features of this enzyme family to aid our strategies to design and develop novel inhibitors with better specificity and lower toxicity and to significantly reduce the possibilities of adverse effects.
